# MicroRNA expression profiling identifies miR-328 regulates cancer stem cell-like SP cells in colorectal cancer

**DOI:** 10.1038/bjc.2012.88

**Published:** 2012-03-27

**Authors:** X T Xu, Q Xu, J L Tong, M M Zhu, F Nie, X Chen, S D Xiao, Z H Ran

**Affiliations:** 1 Division of Gastroenterology and Hepatology, Shanghai Jiao-Tong University School of Medicine Renji Hospital, Shanghai Institute of Digestive Disease, Key Laboratory of Gastroenterology and Hepatology, Ministry of Health (Shanghai Jiao-Tong University), 145 Middle Shandong Road, Shanghai 200001, China

**Keywords:** colorectal cancer, side population cells, cancer stem cell, miR-328, ABCG2, MMP16

## Abstract

**Background:**

Side population (SP) cells and their relationship to stem cell-like properties have been insufficiently studied in colorectal cancer (CRC). MicroRNAs (miRNAs) have attracted much attention but their roles in the maintenance of SP phenotype remain unclear.

**Methods:**

The SPs from CRC cell lines and primary cell cultures were analysed for stem cell-like properties. MiRNA microarray analysis identified miR-328 as a potential stemness miRNA of SP phenotype. The level of miR-328 expression in clinical samples and its correlation with SP fraction were determined. Gain-of-function and loss-of-function studies were performed to examine its roles in cancer stem-like SP cells. Furthermore, bioinformatics prediction and experimental validation were used to identify miR-328 target genes.

**Results:**

The SP cells sorted from CRC possess cancer stem cell (CSC)-like properties, including self-renewal, differentiation, resistance to chemotherapy, invasive and strong tumour formation ability. MiR-328 expression was significantly reduced in SP cells compared with Non-SP cells (*P*<0.05). Moreover, miR-328 expression was downregulated in CRC (*n*=33, *P*<0.05) and low miR-328 expression tend to correlate with high SP fraction (*n*=15, *r*=0.6559, *P*<0.05, Pearson's correlation). Functional studies indicated that miR-328 expression affects the number of SP cells. In addition, miR-328 overexpression reversed drug resistance and inhibited cell invasion of SP cells. Furthermore, luciferase reporter assay demonstrated that miR-328 directly targets ABCG2 and MMP16 and affects the levels of mRNA and protein expression in SP cells.

**Conclusion:**

These findings indicate that CRC contain cancer stem-like SP cells. MiR-328 has an important role in maintaining cancer stem-like SP phenotype that may be a potential target for effective CRC therapy.

Colorectal cancer (CRC) is one of the most commonly diagnosed cancers in both males and females all over the world. It is the second most common cancer in most western countries (Jemal *et al*, 2010) and its incidence is rising in a number of Asian countries during the past few decades (Yee *et al*, 2009). Despite significant improvements have been made in clinical therapy in recent years, most patients still die of this disease as a result of therapeutic resistance and recurrence. Therefore, it is of importance to understand mechanisms of those patients with high recurrence and thus developing approaches to timely intervention.

Recently, an increasing number of studies have revealed the existence of cancer stem cell (CSC) in solid tumours as well as in haematopoietic malignancies, including the CRC (Dalerba *et al*, 2007; O’Brien *et al*, 2007; Ricci-Vitiani *et al*, 2007). It is believed to be responsible for tumour initiation, growth and metastasis (Clarke *et al*, 2006). Relapse of the CRC after intensive therapy including surgery, radiation and chemotherapy may be due to the persistence of CSC.

Side population (SP) cells have been described as a subset of cells highly expressing ABC transporters and exhibiting CSC-like phenotypes. It is a functional assay that can be isolated by fluorescence-activated cell sorting (FACS) techniques based on Hoechst 33342 efflux. The SP cells were first isolated from haematopoietic system (Goodell *et al*, 1996) and more recently from normal tissues and several solid tumours, including neuroblastoma (Hirschmann-Jax *et al*, 2004), nasopharyngeal carcinoma (Wang *et al*, 2007), squamous cell cancers (Loebinger *et al*, 2008; Zhang *et al*, 2009), mesenchymal neoplasm (Wu *et al*, 2007) and bone sarcomas (Murase *et al*, 2009). These studies have suggested that the SP may be a source of CSCs. However, the exact nature of these cells has yet to be elucidated. In the majority of studies, SP cells have been found to be capable of self-renewal, differentiation, resistance to chemotherapeutic agents and tumourigenesis *in vivo* (Moserle *et al*, 2010). However, there are also some studies report that SP cells have not been shown to possess stem cell properties (Triel *et al*, 2004; Burkert *et al*, 2008; Lichtenauer *et al*, 2008; Broadley *et al*, 2011). There have been few studies directly comparing SP and Non-SP in CRC and yielding inconsistent results (Haraguchi *et al*, 2006; Burkert *et al*, 2008). These studies need to be confirmed using more cancer cell lines and especially primary tumour to have a better understanding of the involvement of SP in CRC.

MicroRNAs (miRNAs) are small non-protein coding RNA that acts as master regulators of gene expression through post-transcriptional interactions with mRNA. Emerging evidence demonstrate numerous miRNAs implicated in crucial physiological and pathologic processes such as proliferation (Hime and Somers, 2009), differentiation (Ivey and Srivastava, 2010), apoptosis (Cheng *et al*, 2005) and stem cell self-renewal (Niu *et al*, 2011). Recent researches have shed light on the biological importance of miRNAs in CSC dysregulation (DeSano and Xu, 2009). However, the knowledge of miRNA involvement in SP is scarce, a significant amount of research is needed.

In this study, we applied flow cytometry analysis to identify SP cells in human CRC cell lines and primary tissues. Moreover, we evaluated the biological properties between the SP cells and Non-SP cells. Given the significant role of miRNA in biological processes, we believe that abnormalities of miRNA may contribute to cancer stem-like SP phenotype. We performed miRNA microarray analysis and identified miR-328 as a potential stemness miRNA of SP phenotype. We sought to determine the role of miR-328 in SP phenotype maintenance and test the hypothesis that miR-328 may be targeted in cancer stem-like SP cells of CRC.

## Materials and methods

### Cell lines and clinical samples

The human CRC cell lines SW1116, LoVo, HCT116, SW480 and SW620 were obtained from the Shanghai Institutes for Biological Sciences, Chinese Academy of Sciences, China. The HCPT-resistant cell line SW1116/HCPT was developed in our laboratory using a stepwise concentration-increasing method (Tong *et al*, 2011). SW1116 cells were exposed to an initial HCPT (Tiancheng, Changchun, China) concentration of 0.02 *μ*g ml^−1^ in RPMI-1640 (Invitrogen, Eugene, OR, USA) plus 10% fetal bovine serum (FBS). The surviving population was exposed to the concentration of HCPT gradually with increment of 0.02 *μ*g ml^−1^ (6 months), 0.1 *μ*g ml^−1^ (6 months), and finally to the concentration of 2.4 *μ*g ml^−1^. The surviving resistant cells were named as SW1116/HCPT.

Thirty-three fresh CRC and matched normal colorectal samples were obtained undergoing surgical resection after informed consent in Renji Hospital, Shanghai Jiao-Tong University School of Medicine. The study was approved by the Institutional Review Board. Surgically removed tissues were quickly frozen in liquid nitrogen for miRNA assay. In addition, 15 fresh tumour tissues of them were dissociated and purified to obtain single-cell tumour suspensions either directly used for SP analysis or for *in-vitro* culture as previously described (Cammareri *et al*, 2008). Briefly, fresh resected CRC specimens were minced into small pieces (0.5–1 mm^3^) with sterile scissors and then digested with 1.5 mg ml^−1^ collagenase IV (Invitrogen) and 20 *μ*g ml^−1^ hyaluronidase (Sigma, St Louis, MO, USA) at 37°C into single-cell suspensions. Cells were then resuspended and serially filtered. Clinical information related to corresponding patients is listed in Supplementary Table 1.

### SP analysis using flow cytometry

Fluorescence-activated cell sorting analysis was performed using MoFlo (DakoCytomation, Fort Collins, CO, USA) based on the previously described method with slight modifications (Goodell *et al*, 1996; Golebiewska *et al*, 2011). Briefly, cells were incubated (10^6^ cells per ml) in pre-warmed RPMI-1640 (supplemented with 10% FBS) containing 5 *μ*g ml^−1^ Hoechst 33342 (Sigma) at 37°C for 120 min with intermittent shaking. The control cells were incubated in the presence of 100 *μ*M of verapamil (Sigma). After incubation, cells were centrifuged and resuspended in 1 ml ice-cold PBS supplemented with 2% FBS. In all, 1 *μ*g ml^−1^ propidium iodide (Sigma) was added to label dead cells and then filtered through a 40-*μ*m cell filter (BD Biosciences, Bedford, MA, USA) to obtain single suspension cells. All data were analysed using FlowJo software (Tree Star, Ashland, OR, USA).

### Proliferation and chemosensitivity assay

Freshly sorted SP and Non-SP cells were incubated at 500 cells per well in 96-well plate triplicates and cultured in complete RPMI-1640 to observe the growth rate. During the 6 days, we measured the absorbance of the cells using a tetrazolium salt (WST-8)-based colorimetric assay in the Cell Counting Kit 8 (CCK-8; Dojindo, Kumamoto, Japan). Briefly, 10 *μ*l of CCK-8 solution was added to each well of the plate at specified time points. Then, the plate was incubated for 120 min. Cell viability was determined by scanning with a microplate reader at 450 nm. For the drug sensitivity assay, freshly sorted SP and Non-SP cells were planted at 5000 cells per well in 96-well plates. HCPT or 5-FU (Sigma) was added the following day in a concentration gradient and repeated in four wells. After 72 h of culture, the absorbance of each well was determined with the Bio-Rad enzyme reader (Bio-Rad, Richmond, CA, USA) using CCK-8 assay. The viability rate (VR) was calculated according to the formula: (absorbance of experimental well−absorbance of blank well)/(absorbance of control well−absorbance of blank well) × 100%.

### Soft agar assay and sphere assay

Sorted SP cells or Non-SP cells at 1 × 10^3^ cells per well were plated in six-well plates in complete culture medium (RPMI-1640 with 10% FBS) containing 0.4% agar on top of 0.6% agar in the same medium. To maintain cell viability, overlay additional 1 ml 0.4% agar in culture medium onto each well once a week or when medium start turning yellow. The number of colonies was counted after 3 weeks. The sphere assay was performed as described from a previously published protocol with some modifications. Cells were plated at 500 cells per well in six-well ultra-low attachment plates (Corning Inc., Corning, NY, USA). Sorted SP and Non-SP cells were cultured in serum-free DMEM/F12 medium (Invitrogen) supplemented with fibroblast growth factor (10 ng ml^−1^) and epidermal growth factor (20 ng ml^−1^) (Invitrogen); insulin (25 *μ*g ml^−1^) (Sigma), penicillin (100 units ml^−1^), and streptomycin (100 *μ*g ml^−1^) (Invitrogen). On day 15, the numbers of spheres were counted.

### *In-vitro* differentiation study

To compare the differentiation ability of SP with Non-SP cells *in vitro*, freshly sorted SP and Non-SP cells were cultured separately in RMPI-1640 with 10% FBS. During the 12, 25 and 32 days, both SP and Non-SP cells were stained with Hoechst 33342 and reanalysed the proportion of SP via FACS.

### *In-vitro* invasion assay

Cell invasion assays were conducted using 24-well chambers with 8-*μ*m-pore polycarbonate membranes that were coated with Matrigel (BD Biosciences). Briefly, freshly sorted SP and Non-SP cells were harvested and suspended in serum-free media at a concentration of 5 × 10^5^ per ml. Cells prepared in 100 *μ*l of serum-free media were loaded in the upper wells, and a medium containing 20% FBS was placed in the lower wells as a chemoattractant stimulus. Cell invasion was allowed to proceed for 48 h and non-invading cells were removed with cotton swabs. Invaded cells on the bottom surface of the filter were fixed in 100% methanol for 5 min, stained with 0.1% crystal violet for 10 min. Cells that had invaded through the underside of the inserts were counted by light microscopy.

### Tumourigenicity assay *in vivo*

Eight-week-old male BALB/C nude mice were used for these experiments in accordance with the institutional procedural and ethical guidelines. BALB/C nude mice were supplied by the Shanghai Experimental Animal Center, Chinese Academy of Sciences, Shanghai, China. Various numbers of freshly sorted SP and Non-SP cells (three mice/group) were injected in 200 *μ*l sterile PBS into the axillary fossa of each mouse. Tumour development was monitored starting from the second week. Mice were terminated at 5 weeks after tumour cell injection.

### MiRNA microarray expression profiling and data analysis

We used the illumina sentrix array matrix for miRNA microarray assay according to manufacturer's instructions (Illumina, San Diego, CA, USA). Briefly, total RNA (200 ng) was polyadenylated and reverse transcribed, and the cDNA was hybridised to miRNA bead chip. Sample array matrices (miRNA) were scanned on an illumina Bead Array reader. Microarray data processing and analysis were performed with illumina BeadStudio software.

### qRT–PCR

Total RNA, including miRNA, was isolated using TRIzol reagent according to manufacture’s instruction. SYBR green mRNA qRT–PCR was performed using the primers listed as follows: *ABCG2* (NM_004827.2) sense, 5-CACCTTATTGGCCTCAGGAA-3, and antisense, 5-CCTGCTTGGAAGGCTCTATG-3; *MMP16* (NM_005941.4) sense, 5-CAATTGACTGGATGAAGAAGCCCCG-3, and antisense, 5-AGTGATGTGCTTGTGCTGCCA-3. *GAPDH* (NM_ 002046.3) gene served as internal control, the primers were as follows: sense, 5-GCACCGTCAAGGCTGAGAAC-3, and antisense, 5-ATGGTGGTGAAGACGCCAGT-3.

For miRNA qRT–PCR, expression of mature miR-328 was analysed by TaqMan miRNA Assay (Applied Biosystems, Foster City, CA, USA) under conditions defined by the supplier. The TaqMan probes and primers for human miR-328 and internal control U6 were purchased from Applied Biosystems. Amplification and analysis were performed on the ABI 7900 sequence detection system.

### MiRNA transfection

MiRNAs were designed and synthesised by GenePharma (Shanghai, China). MiRNA transfection was performed using Lipofectamine 2000 (Invitrogen) according to manufacturer’s instructions. Cells were grown in six-well plates to 50% confluence before transfection. Transfections were the following: miR-328 mimic, mimic control, miR-328 inhibitor, inhibitor control and blank control culture medium (Mock).

### Western blotting

Protein extracts were resolved through 10% SDS–PAGE and transferred onto PVDF membranes (Millipore, Billerica, MA, USA), probed with primary antibodies against anti-mouse ABCG2 (Santa Cruz Biotechnology, Santa Cruz, CA, USA), anti-rabbit MMP16 (Epitomics, Burlingame, CA, USA) or GAPDH (Kang Cheng Biotechnology, Shanghai, China) overnight at 4°C. Membranes were incubated with HRP-conjugated anti-mouse or anti-rabbit secondary antibody for 60 min at room temperature, and protein bands were visualised with chemiluminescence detection system (Millipore).

### miRNA luciferase assay

To evaluate the function of miR-328, one putative miR-328-recognition elements from the *ABCG2* gene, two from *MMP16* and corresponding mutants were cloned into the 3′-UTR of the psiCHECK-2 luciferase reporter vector (Promega, San Luis Obispo, CA, USA). All constructs were confirmed by DNA sequence analysis. The oligonucleotides sequences used are shown in Supplementary Table 2. The SP cells sorted from SW1116 cell line, chosen based on their low endogenous expression of miR-328, were co-transfected with mimic control or miR-328 mimic and psiCHECK-2 Dual-Luciferase miRNA target expression vectors using Lipofectamine 2000 (Invitrogen). Luciferase assay was performed using the Dual-Luciferase Reporter Assay System (Promega) at 48 h after transfection. The activity of renilla luciferase was normalised by the internal firefly luciferase activity. All assays were performed in triplicate.

### Statistical analysis

Data presented were mean±s.d. The statistical significance of differences was assessed using unpaired *t*-test (GraphPad Software Inc., La Jolla, CA, USA) for comparison between groups. The association between expression levels of miR-328 and SP fraction was analysed using the Pearson correlation coefficient. *P*-values <0.05 were considered significant.

## Result

### Colorectal tumours contain SP cells

**Figure 1:**
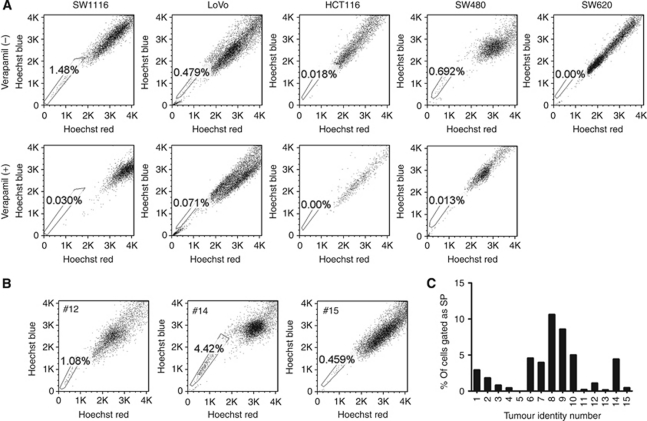
SP cells were present in CRC cell lines and primary CRC samples. CRC cell lines and primary CRC cells were stained with Hoechst 33342 in the presence or absence of 100 *μ*M verapamil and analysed by flow cytometry. Results of five cell lines (SW1116, LoVo, HCT116, SW480 and SW620) (**A**) and three representative surgical resection of CRC samples #12, #14 and #15 were shown (**B**). Percentage of SP found in 15 clinical CRC samples (**C**). The SP cells were gated and shown as the proportion of the whole viable cell population.

To identify and purify SP cells from the CRC, we used fluorescent dye Hoechst 33342 and FACS to detect SP cells in 5 CRC cell lines (SW1116, LoVo, HCT116, SW480 and SW620) and 15 primary CRC samples. Flow cytometry analysis demonstrated that SW1116, LoVo, SW480 and HCT116 cells included SP cells in 1.48%, 0.479%, 0.679% and 0.018%, respectively (Figure 1A). The proportion of SP cells ranged from 0.042% to 10.6% of the total cell populations in primary CRC samples (Figures 1B and C). The SP and Non-SP cells in SW1116, SW480 and three primary CRC samples (#12, #14 and #15) were sorted and applied for further experiments.

### Cell growth curve and anchorage-independent growth of SP and Non-SP cells

**Figure 2:**
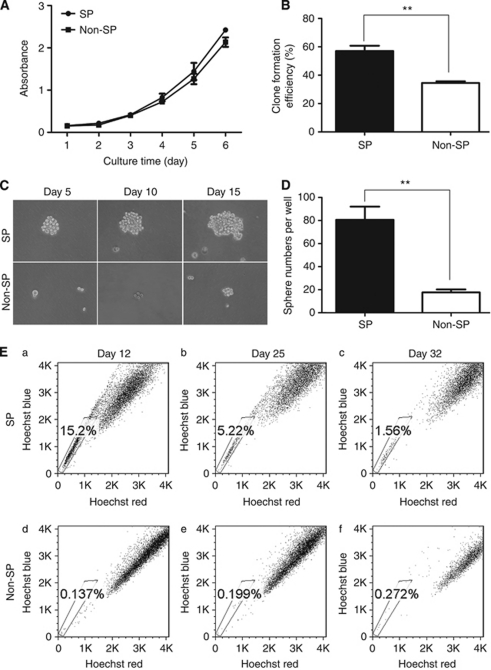
*In-vitro* growth characteristics of SP and Non-SP cells. (**A**) CCK8 assay was performed to determine the cell growth of sorted SP and Non-SP cells cultured in normal RPMI-1640 for 6 days. (**B**) Quantification of soft agar assay of SP and Non-SP cells. The clone formation efficiency at the end of 3 weeks was indicated. (**C**, **D**) Sphere assay of SP and Non-SP cells. Sorted SP and Non-SP cells were seeded into six-well ultra-low attachment plates and cultured in serum-free DMEM/F12 medium. (**C**) Representative images of sphere of sorted SP and Non-SP cells on the fifth day, tenth and fifteenth day are shown. (**D**) The sphere numbers were counted on fifteenth day. (**E**) Differentiation ability of SP and Non-SP cells. SP and Non-SP cells sorted were cultured for 12, 25 and 32 days and then reanalysed using flow cytometry, respectively. The SP generated both a significant SP and Non-SP fraction comparable with the original sort (a, b, c), whereas the Non-SP was mostly Non-SP (d, e, f). Experiments were performed in triplicate with similar results in each case. ^**^P<0.01.

To determine the differences in proliferation, the growth rates of SP and Non-SP cells were measured during second to seventh days after sorting. The data showed that the proliferation rates of the SP cells were not markedly different from the Non-SP cells when cultured in RPMI-1640 with 10% FBS (*P*>0.05; Figure 2A). To explore the difference in ability to form colonies, or spheres, we performed soft agar and sphere assay. Differences between SP and Non-SP cells were quantitated and SP cells showed an increased ability to form colonies under anchorage-independent conditions after 21 days in culture. The SP cells were able to form 1.7 times more colonies than Non-SP cells (*P*<0.05; Figure 2B). Similarly, sphere assay also confirmed that SP cells have an increased number of spheres relative to Non-SP cells (*P*<0.05; Figures 2C and D).

### Differentiation ability of SP and Non-SP cells

To compare the differentiation ability of SP and Non-SP cells, sorted SP and Non-SP cells were further cultured under the serum culture condition *in vitro*. On days 12, 25 and 32, the cells were stained with Hoechst 33342 to reanalyse the SP proportion. We observed that the SP proportion in the SP selected cultures declined rapidly over time and decreased to the proportion presenting in the original cell line when cultured over 32 days. In contrast, the cultured Non-SP cells contained mainly Non-SP cells and the proportion did not change significantly over time (Figure 2E). These results suggest that SP cells, but not Non-SP cells can repopulate both SP and Non-SP cells in culture.

### Drug sensitivity differences between SP and Non-SP cells

**Figure 3:**
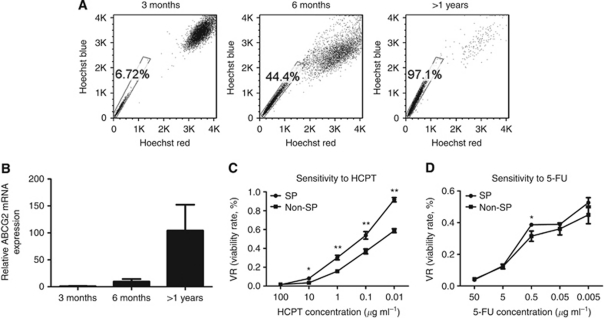
SP cells are more resistant to chemotherapeutic drugs than Non-SP cells. Exposure to HCPT can both increase the SP fraction (**A**) and *ABCG2* gene expression (**B**) by a prolonged selection in SW1116 cells. After the treatment of the chemotherapeutic drugs, HCPT(**C**) or 5-FU (**D**) at the indicated concentrations for 48 h, surviving cells were quantified using CCK8 assays. Each vertical bar represents the mean±s.d. of triplicate determinations. ^*^P<0.05, ^**^P<0.01.

Previous studies revealed that chemotherapy resistance represents the key characteristic of SP cells and ABC transporter especially ABCG2 expression is mainly responsible for this resistance phenotype. We tested whether exposure of SW1116 cells to relevant levels of drug treatment could select for increased proportion of SP cells and ABCG2 expression. In deed, we observed that both the SP fraction (Figure 3A) and *ABCG2* gene expression (Figure 3B) were significantly increased following HCPT-based chemotherapy. A clean SP could be identified and >60-fold increment was showed over the original population by long-term drug exposure. Moreover, we did sensitivity assays and found that SP cells were more resistant to HCPT (*P*<0.05; Figure 3C) and 5-FU (*P*<0.05; Figure 3D), confirming that SP cells are more resistant to chemotherapeutic drugs.

### SP cells were more invasive and tumourigenic

**Figure 4:**
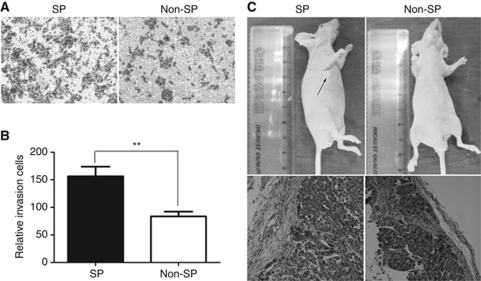
SP cells were more invasive and tumourigenic. (**A**, **B**) The invasion assay was performed using 24-well Matrigel invasion chambers. (**A**) Representative photographs of invading cells. (**B**) Average invasion cells from three independent experiments. (**C**) Freshly sorted SP or Non-SP cells were subcutaneously injected into nude mice and euthanatised 5 weeks later. The H&E staining results of the tumours formed by SP cells (2 × 10^3^) and Non-SP cells (5 × 10^5^), respectively. Each vertical bar represents the mean±s.d. of triplicate determinations. ^**^P<0.01.

Invasion assay was performed to investigate differences in the invasion ability of SP and Non-SP cells *in vitro*. We found SP cells from SW1116 were significantly more invasive than Non-SP cells (*P*<0.05; Figures 4A and B). Furthermore, we investigated the *in-vivo* tumour formation potential of the SP cells. Various numbers of SP and Non-SP cells sorted from SW1116, SW480, #12, #14 and #15 were injected into 8-week-old nude mice subcutaneously and tumour development was monitored for 5 weeks. H&E staining was performed on tumours formed by equal numbers of SP and Non-SP cells when they reached comparable tumour size. As shown in Table 1 and Figure 4C, Non-SP cells could form tumour at 2 × 10^5^ in only one of three mice tested. However, all three mice formed tumours when only 2 × 10^3^ SP cells were inoculated.

### Identification of miRNAs differentially expressed between SP and Non-SP cells

We performed miRNA microarray analysis to investigate the miRNAs differentially expressed in the SP as compared with the Non-SP cells. By using stringent significance criteria of a two-fold or greater difference in expression level and a DiffScore >13 (which equates to a nominal *P*-value <0.05), we found that 73 miRNAs were differentially expressed in SP and Non-SP cells. Of these 73 miRNAs, 33 miRNAs had lower expression and 40 miRNAs had higher expression in SP cells. The complete list of differentially expressed miRNAs between SP and Non-SP cells is provided in Supplementary Table 3. Among the differentially expressed miRNAs, miR-328 attracted our attention. MiR-328 influences drug disposition in human breast cancer cells by downregulation a gene *ABCG2* that has been found to be highly expressed in SP cells. In addition, misexpression of miR-328 has been associated with different types of cancer (Eiring *et al*, 2010; Arora *et al*, 2011), suggesting that deregulation of miR-328 may be involved in SP phenotype. However, the signal pathway(s) regulated by miR-328 and the role of miR-328 in SP phenotype are still largely unknown. Therefore, miR-328 was chosen for further study in greater detail.

### MiR-328 expression is downregulated in CRC and correlates with high SP fraction

**Figure 5:**
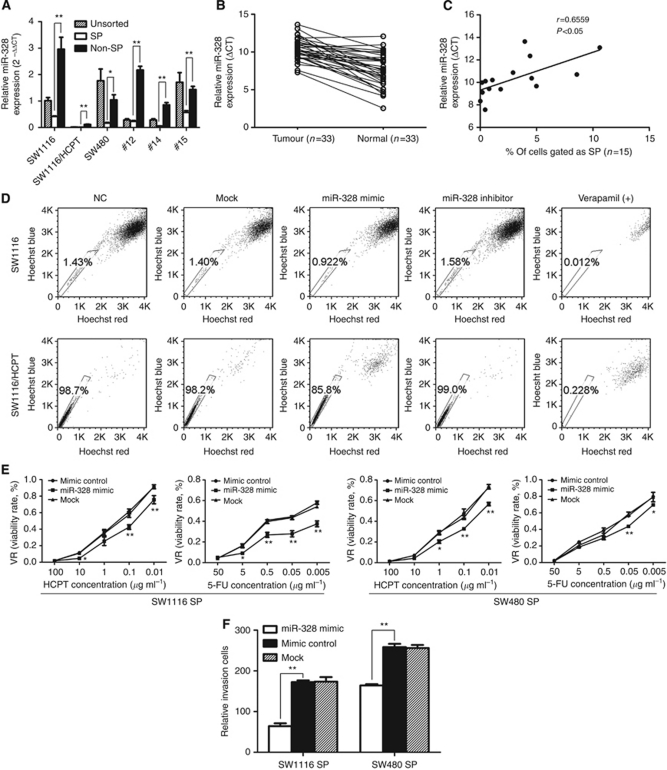
The role for miR-328 in the maintenance of cancer stem-like SP phenotype. (**A**) miR-328 expression in SP and Non-SP cells sorted from SW1116, SW1116/HCPT and SW480 cell lines and three primary CRC cells (#12, #14 and #15). (**B**) miR-328 expression in 33 paired CRC and normal tissue samples. Each individual point on the plot denotes a patient sample. The *y* axis represents the expression of miR-328, which was expressed as the ΔCT (CT miR-328-CT U6). A lower ΔCT indicated higher expression. (**C**) Correlation between miR-328 expression and SP fraction in 15 CRC tissues was analysed by Pearson’s correlation analysis with *r* and *P*-values as indicated (*r*=0.6559, *P*<0.05). (**D**) SW1116 and SW1116/HCPT cells were transfected with NC, mock, miR-328 mimic or miR-328 inhibitor. After 48 h incubation, cells were stained with Hoechst 33342 and analysed by flow cytometry. SP is inhibited by control treatment with verapamil. (**E**, **F**) Sorted SP cells from SW1116 and SW480 cells were transfected with mimic control, mock or miR-328 mimic for 24 h. After reseeding, the drug resistance assay (**E**) and invasion assay (**F**) were performed. Each vertical bar represents the mean±s.d. of triplicate determinations. ^*^P<0.05, ^**^P<0.01.

To validate the results of miRNA array, TaqMan real-time PCR was performed to examine the expression of miR-328. As shown in Figure 5A, SP cells sorted from two cell lines (SW1116 and SW480) and three primary CRC tissues (#12, #14 and #15) revealed significantly lower levels of miR-328 than Non-SP cells. In addition, we also analyse the expression of miR-328 in 33 pairs of CRC specimens and matched normal colorectal tissues, our results showed that the level of miR-328 was reduced in 29 of 33 (87.88%) tumour samples compared with matched normal colorectal tissues. Statistical analysis showed that their mean expression levels (ΔCT) were different (10.38±1.44 *vs* 7.55±2.19, *P*<0.05, Figure 5B). Fifteen fresh tumour tissues for SP analysis were used to further investigate whether the expression of miR-328 (ΔCT) correlates with the proportion of SP fraction. Interestingly, a significant positive correlation was detected (*r*=0.6559, *P*<0.05, Pearson's correlation, Figure 5C). Patients with lower expression of miR-328 (high ΔCT) were found to have significantly higher SP fraction.

### The role for miR-328 in the maintenance of cancer stem-like SP phenotype

To determine if miR-328 is necessary for SP cell phenotype maintenance, we next performed gain-of-function and loss-of-function study to investigate the effects of this miRNA on cancer stem-like SP cells. We compared SW1116 and SW1116/HCPT cells transfected with miR-328 mimic, miR-328 inhibitor or negative control (NC) and cells without transfected (mock) for SP fraction using the flow cytometric analysis. As shown in Figure 5D, a significant inhibition of SP fraction was observed in cells transfected with miR-328 mimic, which exhibited a 36% decrease in SW1116 cells and a 13% decrease in SW1116/HCPT cells as compared with cells transfected with NC (*P*<0.05). Likewise, inhibition of miR-328 increased the SP fraction from 1.43% to 1.58% in SW1116, whereas no significantly change was observed in SW1116/HCPT. The control, verapamil, as expected, also inhibited SP generation. Furthermore, the effect of miR-328 on drug sensitivity and invasion of SP cells sorted from SW1116 and SW480 were investigated. We examined if overexpression of miR-328 is able to override drug resistance. Following 24 h of transfection, sorted SP cells were treated with HCPT or 5-FU for 48 h. Cell viability analysis revealed that miR-328 overexpression reduced cell survival in SP cells (*P*<0.05; Figure 5E). In addition, invasion assay showed that the number of SP cells infected with miR-328 mimic passed through matrigel was significantly reduced as compared with SP cells infected with NC (*P*<0.05; Figure 5F) Taken together, these results support a critical role for miR-328 in the maintenance of cancer stem-like SP Phenotype.

### ABCG2 and MMP16 are target genes for miR-328

**Figure 6:**
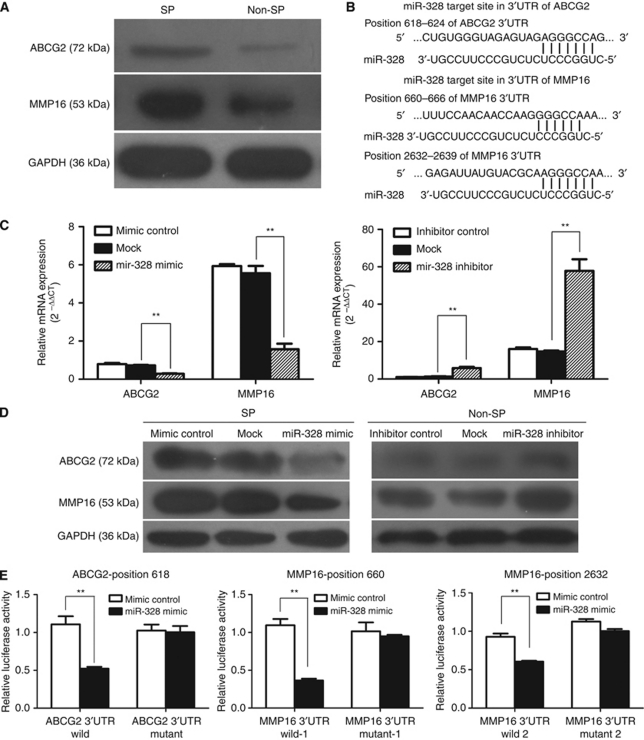
ABCG2 and MMP16 serve as direct targets genes for miR-328. (**A**) Western blot was performed to detect the expression of ABCG2 and MMP16 in SP and Non-SP cells sorted from SW1116 cell line. (**B**) Sequence alignments of miR-328 and its target sites in 3′-UTR of ABCG2 or MMP16 from TargetScan (http://www.targetscan.org) are shown. (**C**, **D**) miR-328 negatively regulated ABCG2 and MMP16 expression in SP and Non-SP cells sorted from SW1116 cell line. qRT–PCR (**C**) and western blot (**D**) for ABCG2 and MMP16 expression in SP cells that were mock transfected or transfected with miR-328 mimic or mimic control (left) and in Non-SP cells that were mock transfected or transfected with miR-328 inhibitor or inhibitor control (right). (**E**) Luciferase reporter assay. SP cells sorted from SW1116 were co-transfected with ABCG2 or MMP16 3′-UTR luciferase reporter plasmid or miR-328 target sites mutated reporter construct, together with mimic control or miR-328 mimic as indicated. After 48 h, firefly and renilla luciferases were measured in cell lysates. The activity of renilla luciferase was normalised by the internal firefly luciferase activity. ^**^P<0.01.

Next, we sought to investigate the mechanism responsible for the effect of miR-328 on SP phenotype observed above. Our western bolt results showed that the levels of ABCG2 and MMP16 protein were significantly higher in SP cells compared with Non-SP cells (Figure 6A). ABCG2, which is a member of ATP-binding cassette transporters, has been recently reported as a target gene for miR-328 in breast cancer cell lines. In addition, we found that human MMP16, known to have critical roles in cancer invasion, contained putative conserved miR-328 target site (Figure 6B). To examine whether miR-328 affects ABCG2 and MMP16 expression by targeting the 3′-UTR, we employed a gene overexpression and knockdown approach in SP cells (which expressed low level of miR-328) and Non-SP cells (which expressed high level of miR-328) sorted from SW1116 cell line, respectively. As qRT–PCR and western blot analysis shown in Figures 6C and D, both ABCG2 and MMP16 expressions were inversely correlated with miR-328 expression in SP and Non-SP cells. To further confirm ABCG2 and MMP16 as direct targets of miR-328, we constructed luciferase reporter plasmids containing the miR-328 recognition site or a mutated sequence at this site cloned from the 3′-UTR of *ABCG2* mRNA or *MMP16* mRNA. As shown in Figure 6E, transfection of SP cells with miR-328 mimic suppressed the luciferase activity of the reporter vectors containing the 3′-UTR of wild-type ABCG2 or wild-type MMP16, but failed to inhibit that of the target site-mutated construct by dual-luciferase reporter assay. Taken together, these data indicate that miR-328 downregulation may contribute to the overexpression of ABCG2 and MMP16 in SP cells.

## Discussion

In recent years, it has been shown that SP cells exist in various types of tumours, including the CRC (Haraguchi *et al*, 2006; Sussman *et al*, 2007; Burkert *et al*, 2008). However, it is important to note that these studies are conflicting and stringent functional characterisation of SP cells in CRC is unclear. In the current study, we demonstrated that CRC cell lines and primary tissues contained cancer stem-like SP cells. Furthermore, we found that miR-328 expression is downregulated in CRC and correlates with high SP fraction. MiR-328 has an important role in maintaining cancer stem-like SP phenotype by directly targets ABCG2 and MMP16.

Our present data are consistent with previous studies, which showed that SP cells isolated from CRC have many CSC properties, including self-renewal, differentiation, resistance to chemotherapy and strong tumour formation ability *in vivo*. Moreover, our *in-vitro* invasion assay found that SP cells were more invasive than Non-SP suggests that SP cells may be related to CRC invasiveness. There has been conflicting data regarding the toxicity of Hoechst 33342 dye to live cells (Montanaro *et al*, 2004; Platet *et al*, 2007). Our result showed that SP cells have almost similar proliferation rate as Non-SP cells, coupled with previous work (Huang *et al*, 2009; Song *et al*, 2010), suggesting that cytotoxicity does not account for the cancer stem-like phenotype of SP cells.

Some recent studies revealed that cellular stresses like drug exposure (Chua *et al*, 2008; Calcagno *et al*, 2010; Fong *et al*, 2010), hypoxia (Bar *et al*, 2010) and UV lighting (Liang *et al*, 2010) enriched for a CSC phenotype in cancer cell lines. In this study, we detected 1% cells positive for SP in the primary line SW1116 and observed a remarkable increase to 6%, 44% and 97% after HCPT treatment to 3 months, 6 months and >1 years, respectively. More interestingly, *ABCG2* gene expression also increased consistent with SP enrichment by drug selection. Our data suggested that SP can be highly enriched by drug treatment and ABCG2 seem to strongly correlate with maintenance of SP features.

Emerging evidence suggests that miRNA has important roles in physiologic processes and aberrant expression could contribute to the initiation and progression of cancer (Calin and Croce, 2006). Functional studies of the specific miRNAs within CSCs, specifically SP cells, are necessary to find a therapeutic target. The present study determined that miR-328 was significantly downregulated in CRC tissues, and that this downregulation was correlated with high SP fraction. In addition, aberrant expression of miR-328 contributes to the cancer stem-like phenotypes of SP cells, such as drug resistant and cell invasion. However, underlying mechanisms that mediate the downregulation of miR-328 in SP cells have not been elucidated. Our results confirmed that miR-328 directly targets ABCG2, which is in agreement with recent studies (Pan *et al*, 2009; Li *et al*, 2010), supporting that miR-328 affect the number of SP cells and drug resistant in part through this pathway. In addition to ABCG2, we found that MMP16 is also a direct target of miR-328. MMP16 is one member of the matrix metalloproteinase (MMP) family and can degrade type III collagen, gelatin, fibronectin and laminin-1 (Kang *et al*, 2000). Previous studies have shown that MMP16 enhance the growth and invasiveness of Madin-Darby canine kidney cells (Kitagawa *et al*, 1999) and gastric cancer cells (Lowy *et al*, 2006). The invasion inhibitory effect of miR-328 is probably related to this target. MiR-328 may be a critical small non-coding RNA with potential to specific target cancer stem-like SP cells and could be a new strategy for CRC treatment. Obviously, other undisclosed miR-328 targets may contribute to the phenotypic effects observed on cancer stem-like SP cells. The involvement of other potential targets should be elucidated in future studies.

In summary, the present study provides evidence that SP cells are enriched CSCs in CRC. We performed miRNA expression profiles and identified a set of differentially expressed miRNAs between SP cells and Non-SP cells. Our study highlights the importance of miR-328 for the maintenance of SP phenotype. Future work to identify the gene and protein networks directly targeted and affected and the entire roles of miR-328 in SP phenotype remain to be explored.

## Figures and Tables

**Table 1:** *In-vivo* tumour formation potential of the SP cells and Non-SP cells

	**Cell no. (weeks)**				
	**1 × 10^6^ (5)**	**2 × 10^5^ (5)**	**2 × 10^4^ (5)**	**2 × 10^3^ (5)**	**2 × 10^2^ (5)**
SW1116 SP	—	3/3	3/3	3/3	0/3
Non-SP	2/3	1/3	0/3	0/3	0/3
SW480 SP	—	2/3	3/3	3/3	1/3
Non-SP	1/3	1/3	0/3	0/3	0/3
#12 SP	3/3	1/3	0/3	—	—
Non-SP	0/3	0/3	0/3	—	—
#14 SP	3/3	2/3	1/3	—	—
Non-SP	0/3	0/3	0/3	—	—
#15 SP	2/3	1/3	0/3	—	—
Non-SP	0/3	0/3	0/3	—	—

SP and Non-SP cells were isolated separately and injected subcutaneously into BALB/C mice. Tumour formation was observed for 5 weeks after injection.
