# Supplementary material for: MicroRNA expression profiling identifies miR-328 regulates cancer stem cell-like SP cells in colorectal cancer
Source: Br J Cancer. 2012 Mar 27;106(7):1320–30. doi: 10.1038/bjc.2012.88 (PMC3314795; doi:10.1038/bjc.2012.88)
Supplement: Supplementary Information [file bjc201288x1.doc]

### Supplementary online material

**Supplementary Table 1 Characteristics of patients used** in this study

| Patient number | Age/sex | Tumor site | TNM stage | SP (%) |
| --- | --- | --- | --- | --- |
| #1 | 79/M | Rectal | T_3_N_0_M_1_ | 2.91 |
| #2 | 72/M | Sigmoid | T_3_N_0_M_1_ | 1.81 |
| #3 | 55/M | Rectal | T_4_N_1_M_0_ | 0.799 |
| #4 | 77/M | Rectal | T_3_N_0_M_0_ | 0.434 |
| #5 | 56/M | Rectal | T_4_N_2_M_0_ | 0.042 |
| #6 | 78/F | Sigmoid | T_4_N_0_M_0_ | 4.56 |
| #7 | 54/M | Rectal | T_4_N_1_M_0_ | 3.96 |
| #8 | 60/F | Sigmoid | T_3_N_2_M_0_ | 10.6 |
| #9 | 81/F | Right colon | T_3_N_2_M_1_ | 8.57 |
| #10 | 63/F | Rectal | T_4_N_1_M_1_ | 4.98 |
| #11 | 72/F | Right colon | T_3_N_0_M_0_ | 0.195 |
| #12 | 56/M | Rectal | T_1_N_0_M_0_ | 1.08 |
| #13 | 73/M | Rectal | T_1_N_1_M_1_ | 0.162 |
| #14 | 86/M | Sigmoid | T_3_N_1_M_0_ | 4.42 |
| #15 | 35/M | Rectal | T_3_N_1_M_0_ | 0.459 |
| #16 | 68/M | Rectal | T_3_N_0_M_0_ | - |
| #17 | 34/F | Rectal | T_3_N_1_M_0_ | - |
| #18 | 57/F | Right colon | T_3_N_1_M_0_ | - |
| #19 | 57/F | Left colon | T_3_N_1_M_0_ | - |
| #20 | 54/M | Rectal | T_3_N_2_M_0_ | - |
| #21 | 82/M | Left colon | T_3_N_1_M_0_ | - |
| #22 | 65/F | Rectal | T_2_N_0_M_0_ | - |
| #23 | 82/F | Rectal | T_3_N_0_M_0_ | - |
| #24 | 82/F | Right colon | T_3_N_0_M_0_ | - |
| #25 | 58/M | Rectal | T_3_N_0_M_0_ | - |
| #26 | 78/M | Rectal | T_1_N_0_M_0_ | - |
| #27 | 59/F | Right colon | T_3_N_0_M_0_ | - |
| #28 | 59/F | Right colon | T_3_N_1_M_0_ | - |
| #29 | 72/M | Rectal | T_4_N_2_M_1_ | - |
| #30 | 74/M | Right colon | T_3_N_0_M_0_ | - |
| #31 | 60/M | Rectal | T_3_N_1_M_1_ | - |
| #32 | 53/M | Right colon | T_3_N_1_M_0_ | - |
| #33 | 46/M | Rectal | T_3_N_0_M_0_ | - |

**Supplementary Table 2 The oligonucleotides sequences used as inserts for 3’UTR luciferase assay**

| name | Sequences |
| --- | --- |
| ABCG2-wild type-S | 5′GGGTTTGGAACTGTGGGTAGAGTAGAGGGCCAGGAGTCCAGTA3′ |
| ABCG2-wild type-A | 5′TACTGGACTCCTGGCCCTCTACTCTACCCACAGTTCCAAACCC3′ |
| ABCG2-mutant type-S | 5′GGGTTTGGAACTGTGGGTAGAGTAGTCCCGGTGGAGTCCAGTA3′ |
| ABCG2-mutant type-A | 5′TACTGGACTCCACCGGGACTACTCTACCCACAGTTCCAAACCC3′ |
| MMP16-wild type 1-S | 5′TGTTGGGACAGCCATTTTCCAACAACCAAGGGGCCAAAATATCTGCAATATAG3′ |
| MMP16-wild type 1-A | 5′CTATATTGCAGATATTTTGGCCCCTTGGTTGTTGGAAAATGGCTGTCCCAACA3′ |
| MMP16-mutant type 1-S | 5′TGTTGGGACAGCCATTTTCCAACAACCAAGCCCGGTTAATATCTGCAATATAG3′ |
| MMP16-mutant type 1-A | 5′CTATATTGCAGATATTAACCGGGCTTGGTTGTTGGAAAATGGCTGTCCCAACA3′ |
| MMP16-wild type 2-S | 5′ATTTTAAGAGGATATTGGAGATTATGTACGCAAGGGCCAAGAAAGCAAGAAATGAGA3′ |
| MMP16-wild type 2-A | 5′TCTCATTTCTTGCTTTCTTGGCCCTTGCGTACATAATCTCCAATATCCTCTTAAAAT3′ |
| MMP16-mutant type 2-S | 5′ATTTTAAGAGGATATTGGAGATTATGTACGCATCCCGGTTGAAAGCAAGAAATGAGA3′ |
| MMP16-mutant type 2-A | 5′TCTCATTTCTTGCTTTCAACCGGGATGCGTACATAATCTCCAATATCCTCTTAAAAT′3′ |

**Supplementary Table 3 MicroRNAs differentially expressed between SP and Non-SP cells**

| ILMN_GENE | PROBE_ID | FOLD CHANGE  (Non-SP /SP) | DIFFSCORE | DIFF PVAL |
| --- | --- | --- | --- | --- |
| hsa-miR-654-3p | ILMN_3168801 | 35.37 | -82.3044 | 5.88E-09 |
| hsa-miR-302d | ILMN_3167386 | 24.29 | -107.647 | 1.72E-11 |
| hsa-miR-622 | ILMN_3167141 | 21.30 | -153.884 | 4.09E-16 |
| hsa-miR-18b* | ILMN_3168840 | 21.08 | -76.303 | 2.34E-08 |
| hsa-miR-632 | ILMN_3168336 | 20.32 | -106.957 | 2.02E-11 |
| hsa-miR-346 | ILMN_3168451 | 14.60 | -113.142 | 4.85E-12 |
| hsa-miR-149* | ILMN_3168611 | 13.63 | -87.429 | 1.81E-09 |
| hsa-miR-938 | ILMN_3168842 | 13.25 | -136.228 | 2.38E-14 |
| hsa-miR-302b* | ILMN_3168322 | 9.10 | -120.32 | 9.29E-13 |
| hsa-miR-494 | ILMN_3168446 | 8.83 | -116.009 | 2.51E-12 |
| hsa-miR-518b | ILMN_3167241 | 7.28 | -40.5991 | 8.71E-05 |
| hsa-miR-504 | ILMN_3168485 | 6.84 | -132.459 | 5.68E-14 |
| hsa-miR-220b | ILMN_3168674 | 5.71 | -29.9541 | 0.00101062 |
| hsa-miR-517a/b | ILMN_3167132 | 5.65 | -31.2227 | 0.000754622 |
| hsa-miR-610 | ILMN_3167193 | 4.48 | -26.1599 | 0.00242109 |
| hsa-miR-199a/b-3p | ILMN_3168576 | 4.01 | -25.6512 | 0.002721923 |
| hsa-miR-423-5p | ILMN_3168835 | 3.93 | -77.7664 | 1.67E-08 |
| hsa-miR-1234 | ILMN_3168821 | 3.77 | -39.2407 | 0.000119105 |
| hsa-miR-1238 | ILMN_3168737 | 3.70 | -70.2912 | 9.35E-08 |
| hsa-miR-923 | ILMN_3168768 | 3.53 | -46.0434 | 2.49E-05 |
| hsa-miR-15a* | ILMN_3168662 | 3.52 | -55.0323 | 3.14E-06 |
| hsa-miR-1254 | ILMN_3168605 | 3.51 | -73.993 | 3.99E-08 |
| hsa-miR-223 | ILMN_3166979 | 3.38 | -44.9554 | 3.19E-05 |
| hsa-miR-451 | ILMN_3167614 | 3.05 | -36.6385 | 0.000216844 |
| hsa-miR-1225-3p | ILMN_3168832 | 3.05 | -45.5485 | 2.79E-05 |
| hsa-miR-1290 | ILMN_3168851 | 2.94 | -40.306 | 9.32E-05 |
| hsa-miR-603 | ILMN_3167848 | 2.93 | -22.9664 | 0.005050829 |
| hsa-miR-328 | ILMN_3168198 | 2.93 | -29.4056 | 0.001146665 |
| hsa-miR-199a*:9.1 | ILMN_3168478 | 2.90 | -43.6476 | 4.32E-05 |
| hsa-miR-296-3p | ILMN_3168049 | 2.84 | -18.9103 | 0.01285201 |
| hsa-miR-9* | ILMN_3167194 | 2.58 | -14.5681 | 0.03492962 |
| hsa-miR-663 | ILMN_3167088 | 2.26 | -24.953 | 0.003196705 |
| hsa-miR-551a | ILMN_3168265 | 2.24 | -32.6532 | 0.000542848 |
| hsa-miR-550* | ILMN_3168324 | 0.61 | 18.65029 | 0.01364493 |
| hsa-miR-200a | ILMN_3167801 | 0.50 | 14.86206 | 0.03264332 |
| hsa-miR-339-3p | ILMN_3168833 | 0.49 | 14.68588 | 0.03399473 |
| hsa-miR-769-5p | ILMN_3167148 | 0.49 | 27.86266 | 0.001635813 |
| hsa-miR-340* | ILMN_3166998 | 0.48 | 17.55985 | 0.01753942 |
| hsa-miR-548e | ILMN_3168533 | 0.46 | 13.97583 | 0.04003292 |
| hsa-miR-582-3p | ILMN_3168780 | 0.45 | 29.56383 | 0.001105649 |
| hsa-miR-340 | ILMN_3168866 | 0.45 | 24.59927 | 0.003467951 |
| hsa-miR-141 | ILMN_3168064 | 0.45 | 23.60016 | 0.004365001 |
| hsa-miR-542-5p | ILMN_3167175 | 0.44 | 29.26077 | 0.001185559 |
| hsa-miR-1250 | ILMN_3168585 | 0.44 | 27.50439 | 0.001776482 |
| hsa-miR-219-5p | ILMN_3167523 | 0.44 | 26.53156 | 0.002222512 |
| hsa-miR-521 | ILMN_3168215 | 0.44 | 25.86102 | 0.002593573 |
| hsa-miR-744* | ILMN_3168733 | 0.44 | 13.18528 | 0.04802557 |
| hsa-miR-32* | ILMN_3168655 | 0.43 | 13.79223 | 0.04176156 |
| hsa-miR-188-5p | ILMN_3167745 | 0.43 | 22.34124 | 0.005832791 |
| hsa-let-7i* | ILMN_3168724 | 0.43 | 39.24803 | 0.000118904 |
| hsa-miR-497 | ILMN_3167437 | 0.43 | 32.00114 | 0.000630792 |
| hsa-miR-1287 | ILMN_3168848 | 0.42 | 27.23847 | 0.001888656 |
| hsa-miR-519a | ILMN_3168168 | 0.41 | 30.66244 | 0.000858531 |
| hsa-miR-148b* | ILMN_3168567 | 0.40 | 25.61888 | 0.00274228 |
| hsa-miR-338-5p | ILMN_3168552 | 0.40 | 15.24916 | 0.02985962 |
| hsa-miR-16-1* | ILMN_3168676 | 0.39 | 20.45879 | 0.008997491 |
| hsa-miR-545:9.1 | ILMN_3168175 | 0.36 | 25.82479 | 0.002615295 |
| hsa-miR-518a-5p,  hsa-miR-527 | ILMN_3168499 | 0.36 | 17.4867 | 0.01783734 |
| hsa-miR-216a | ILMN_3167355 | 0.35 | 32.92799 | 0.000509567 |
| hsa-miR-873 | ILMN_3167953 | 0.35 | 26.64286 | 0.002166278 |
| hsa-miR-33a | ILMN_3167691 | 0.35 | 51.81409 | 6.59E-06 |
| hsa-miR-183* | ILMN_3167593 | 0.34 | 19.58256 | 0.01100889 |
| hsa-miR-890 | ILMN_3168791 | 0.32 | 18.60305 | 0.01379416 |
| hsa-miR-570 | ILMN_3167815 | 0.31 | 30.54581 | 0.000881899 |
| hsa-miR-19b-1* | ILMN_3168622 | 0.30 | 40.10516 | 9.76E-05 |
| hsa-miR-193a-3p | ILMN_3168366 | 0.29 | 48.1841 | 1.52E-05 |
| hsa-miR-148a* | ILMN_3168549 | 0.29 | 20.14202 | 0.009678282 |
| hsa-miR-1537 | ILMN_3167659 | 0.29 | 19.19423 | 0.01203863 |
| hsa-miR-19a* | ILMN_3168624 | 0.29 | 21.27813 | 0.007450525 |
| hsa-miR-449a | ILMN_3167451 | 0.28 | 27.53446 | 0.001764226 |
| hsa-miR-147b | ILMN_3168776 | 0.26 | 30.72487 | 0.000846278 |
| hsa-miR-573 | ILMN_3167242 | 0.25 | 21.3258 | 0.007369188 |
| hsa-miR-551b | ILMN_3166993 | 0.25 | 27.01178 | 0.001989859 |
| hsa-miR-101* | ILMN_3168196 | 0.16 | 32.30139 | 0.000588655 |
